# The 21st century water quality challenges for managed aquifer recharge: towards a risk-based regulatory approach

**DOI:** 10.1007/s10040-022-02543-z

**Published:** 2022-09-27

**Authors:** Yan Zheng, Joanne Vanderzalm, Niels Hartog, Enrique Fernández Escalante, Catalin Stefan

**Affiliations:** 1 School of Environmental Science and Engineering, Southern University of Science and Technology, Shenzhen, 518055 China; 2 CSIRO Land and Water, Waite Road, Urrbrae, South Australia 5064 Australia; 3 KWR Water Research Institute, Groningenhaven 7, 3433 PE Nieuwegein, the Netherlands; 4 Grupo Tragsa, Maldonado 58, 28006 Madrid, Spain; 5 Research Group INOWAS, Technische Universität Dresden, 01062 Dresden, Germany

**Keywords:** Water-resources management, Managed aquifer recharge (MAR), Groundwater sustainability, Water quality risks, Attenuation zone

## Abstract

Sustained environmental and human health protection is threatened by ~350,000 chemicals available in global markets, plus new biological entities including coronaviruses. These water-quality hazards challenge the proponents of managed aquifer recharge (MAR) who seek to ensure the integrity of groundwater. A risk-based regulatory framework accounting for groundwater quality changes, adoption in subsurface attenuation zones, and use of advanced monitoring methods is required to support confidence in the sustainability of MAR.

## Résumé

La protection durable de l’environnement et de la santé humaine est menacée par ~350,000 substances chimiques disponibles sur les marchés mondiaux, à quoi s’ajoutent de nouvelles entités biologiques, incluant les coronavirus. Ces risques sur la qualité de l’eau sont un défi pour les partisans de la recharge raisonnée des aquifères (RRA) qui cherchent à assurer l’intégrité des eaux souterraines. Un cadre réglementaire basé sur les risques, tenant compte des changements de qualité des eaux souterraines, de l’adoption de zones d’atténuation souterraine, de l’utilisation de méthodes de contrôle avancées, est nécessaire pour soutenir la confiance dans la durabilité de la RRA.

## Resumen

La sostenibilidad del medio ambiente y la protección de la salud humana se ve amenazada por unas 350,000 sustancias químicas disponibles en los mercados mundiales, además de nuevas entidades biológicas como el coronavirus. Estos peligros para la calidad del agua suponen un desafío para los defensores de la recarga gestionada de acuíferos (MAR), que pretenden garantizar la integridad de las aguas subterráneas. Se necesita un marco normativo basado en el riesgo que tenga en cuenta los cambios en la calidad de las aguas subterráneas, la incorporación a las zonas de atenuación del subsuelo y el uso de métodos avanzados de control para respaldar la confianza en la sostenibilidad de la recarga acuífera gestionada.

## Abstract (zh)

全球约350,000种市售化学品以及包括冠状病毒在内的新污染物严重威胁了人体健康和生态安全，是21世纪地下水人工回补全球应用的重大挑战。因此，基于多种含水层人工调控技术的回补工程在补充水量的同时，需针对各类新旧水污染物，制定补径排全过程风险管控的法规和因地制宜的技术导则。本文强调需推进含水层分区、分层管理，以及划定污染物地下衰减、监控区域的法规制度，充分发挥含水层自净能力，以实现水资源的高效循环和可持续利用。

## Resumo

A proteção sustentável do meio ambiente e da saúde humana está ameaçada por cerca de 350,000 produtos químicos disponíveis nos mercados globais, além de novas entidades biológicas, incluindo coronavírus. Esses riscos de qualidade da água desafiam os proponentes da recarga gerenciada de aquíferos (RGA) que buscam garantir a integridade das águas subterrâneas. Uma estrutura regulatória baseada em risco que considere as mudanças na qualidade das águas subterrâneas, a adoção em zonas de atenuação do subsolo e o uso de métodos avançados de monitoramento são necessários para apoiar a confiança na sustentabilidade da RGA.

## Introduction

Managed aquifer recharge (MAR) is the *purposeful* recharge of water to aquifers for subsequent recovery or environmental benefit (IAH 2022). A rigorous environmental and social sustainability assessment of 28 schemes from 21 countries demonstrates that MAR is a sustainable technology (Zheng et al. 2021). This nature-based engineering approach is poised to play an increasingly significant role in climate change adaptation through augmenting water supply and environmental flows, and recycling treated wastewater. This essay calls upon hydrogeologists worldwide to rise to the 21st-century water-quality challenges using MAR, to maintain the integrity of groundwater resources and to meet humanity’s demand for good quality freshwater.

**Fig. 1:**
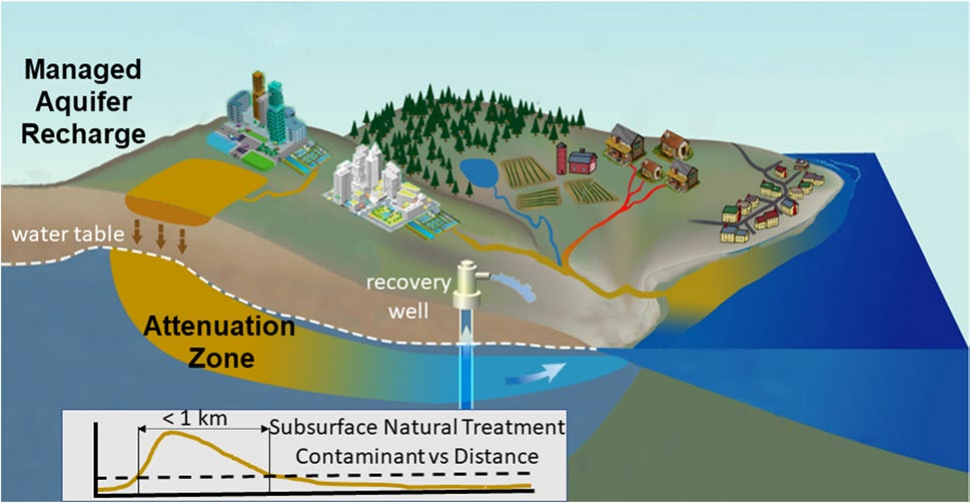
A schematic diagram illustrating how, with the example of an infiltration pond, MAR has been used to purify purposefully recharged water through a series of natural treatment processes occurring in the unsaturated and saturated zones of an aquifer to facilitate the removal of organic pollutants and pathogenic microbes. Here, it is recommended that an attenuation zone (after NRMMC, EPHC, NHMRC 2009) is defined as an independent regulatory unit so that groundwater quality beyond this zone is sustainably protected. Note that the diagram is not to scale because the attenuation zone is usually confined beneath the land owned by the MAR operator and normally <50–300 m

Here it is argued that “novel entities” (NE), defined as “new substances, new forms of existing substances and modified life forms” (Steffen et al. 2015), need to be considered. These NEs include “chemicals and other new types of engineered materials or organisms not previously known to the Earth system as well as naturally occurring elements (for example, heavy metals) mobilized by anthropogenic activities” (Steffen et al. 2015). Considering NEs means that proponents of MAR must go beyond managing risks associated with known legacy pollutants, such as hydrocarbons, pesticides, and disinfection by-products, which can amount to several hundred regulated water quality parameters (Escalante et al. 2020). It also requires addressing not (yet) regulated, and sometimes novel (unknown) water quality threats. Clearly, the capacity to manage current, emerging, and unforeseen water quality risks is critical and relies upon chemical and biological reactions to “purify” purposefully recharged water within a subsurface attenuation zone, a concept originating in Australia (Fig. 1). To gain regulatory approval for this attenuation zone, MAR practitioners have had to demonstrate, using laboratory and field monitoring, the aquifer’s treatment capacity and protection of the aquifer’s groundwater environmental values beyond the attenuation zone. However, regulators may still be inclined to regard the subsurface environment as “pristine” and which should not be “disturbed” by any means. In reality, the interaction of “unmanaged” recharge with a wide range of anthropogenic activities has led to widespread groundwater quality decline. This call to action begins with a historical perspective on water quality issues frequently encountered in MAR. Then, a resolution to tackle this challenge to ensure sustained MAR implementation globally is discussed.

## Historical perspective

**Fig. 2:**
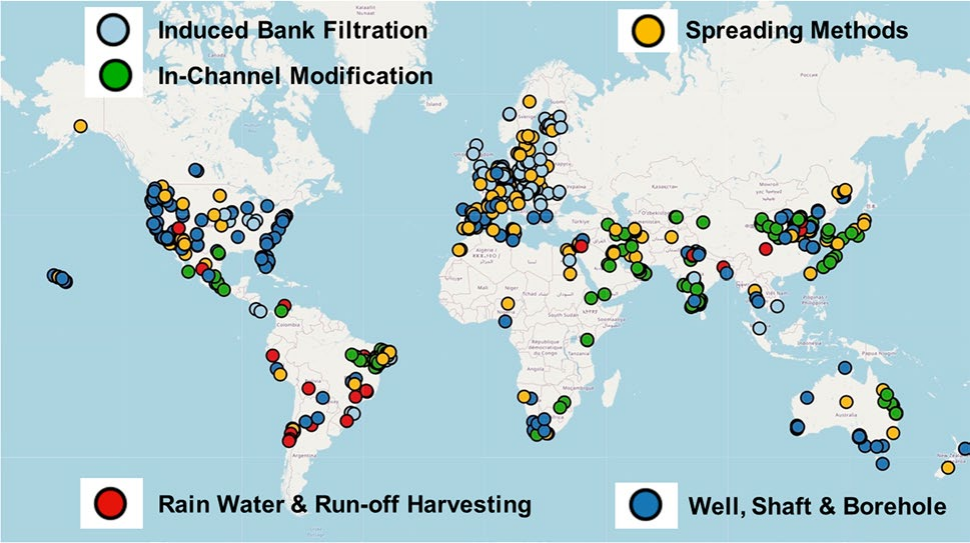
Global inventory of MAR schemes presented as an online portal with the database being continuously updated (IGRAC 2022)

An account of 60 years of global progress of MAR estimated that purposeful recharge has reached 10 km^3^/year, ~2.4% of groundwater extraction in countries reporting MAR, or ~1.0% of global groundwater extraction (Dillon et al. 2019). A global inventory of MAR, including 1,136 pilot and full-scale MAR schemes from 60 countries (Fig. 2, Stefan and Ansems 2018) found that the influent water source, the main objective of the scheme, and the final use of recovered water were well reported (96, 82 and 73% of the total number of cases, respectively). Although a detailed assessment of water quality (over 100 considered) was available in less than 5% of cases, water quality changes were mentioned in many papers, especially in conference papers and specific technical reports.

The important role of water quality investigations in MAR is illustrated by a search of the Science Citation Index (SCI) Expanded database (period from year 1900 to 12 February 2022) using combined topics of MAR and artificial recharge (AR, as it was widely used in the past), with and without ‘water quality’ as a topic. Just above one-third of publications, or 118 out of 391 papers, included water quality, and this proportion remained fairly constant through the years. This is consistent with presentations made at recent International Symposiums on Managed Aquifer Recharge (ISMAR, in 2016 and 2019) where ~34% mentioned water quality (125 out of 371 papers). Presenters at ISMAR conferences (many MAR practitioners do not publish SCI papers) acknowledge that water quality is one aspect to be considered during MAR planning, construction, and operation. Studies aimed at improving understanding of processes regulating water quality and clogging, managing potential degradation or enhancing treatment, are pursued with vigor. Comprehensive laboratory and field-scale experiments which provide sufficient data for reactive transport modelling (e.g., (Prommer and Stuyfzand 2005)) have been invaluable in elucidating the controlling processes and developing management strategies as required. This advancement has led to a recent focus on strategies to optimize water quality treatment, such as through advanced pre-treatment or by a combination of MAR types (Hellauer et al. 2018), incorporation of reactive barriers (Valhondo et al. 2020), or manipulation of the subsurface redox zones (Bartak et al. 2017).

Water quality investigations during MAR projects serve multiple aims. While monitoring for regulatory compliance is a basic starting point, it is not sufficient to adequately manage water quality. Here inorganic arsenic is used to illustrate the importance of having a good understanding of hydrogeochemical processes and their potential impact on MAR operations, with the ability to make prediction a plus. Arsenic is such a highly toxic chemical that even the regulatory limit of 10 μg/L adopted by most countries is not entirely protective of public health. In Florida (USA), injecting oxygenated Tampa City supply water into the Suwannee Limestone of the Upper Floridan aquifer containing pyrite (Price and Pichler 2006) resulted in pyrite-oxidation-driven arsenic release, with recovered water arsenic concentrations frequently exceeding 10 μg/L and rising to as high as 130 μg/L (Jones and Pichler 2007). Recharging a reduced, As-rich coastal aquifer in Khulna, Bangladesh, with pond water resulted in arsenic concentrations in recovered water of >100 μg/L (Sultana et al. 2015) and was attributed to reductive dissolution of As-bearing Fe-oxyhydroxide. A recent critical review on mobilization of arsenic and other naturally occurring contaminants during MAR (Fakhreddine et al. 2021) concludes that arsenic poses the most widespread challenge at MAR sites due to its ubiquity in subsurface sediments and toxicity at trace concentrations; other geogenic contaminants of concern include iron, molybdenum, manganese, chromium, and fluoride. Fortunately, the same review points out many approaches to mitigate MAR-induced arsenic problems in recovered water, but these need process understanding and predictive capability to ensure such risks are managed appropriately. A key step in prediction is an early stage hydrogeochemical investigation to characterize the aquifer and source of water for recharge for conceptual understanding of geochemical reactions and their potential impact (or risk).

Furthermore, risk-based management is essential for the future of MAR (Imig et al. 2022), to ensure the protection of public and environmental health, while also fully utilizing the potential of MAR to provide natural treatment and facilitate recycling and reuse (Fig. 1). Increasing reliance on multiple source waters (e.g., agricultural return flow, urban storm water, and reclaimed water) also expands complexity in water-quality-risk management and regulations for MAR, making the designation of an attenuation zone in MAR regulation ever more relevant (Fig. 1). Such complex and uncertain risks can be dealt with through decades of experience in water quality improvement and management in MAR, furthered by targeted research—for example, a study that evaluates the die-off of plant pathogenic bacteria when stormwater is used to recharge a brackish anoxic aquifer in the Netherlands can enhance confidence in the recovered water’s intended use for irrigation (Eisfeld et al. 2021). Knowledge of biodegradation of trace organic chemicals or contaminants of concern has been advanced through the application of genomic markers to infer the prevailing trophic state of microbial communities in a MAR scheme, and subsequently, predict favorable conditions for removal (Filter et al. 2021). While it is understood that microbially mediated processes are an important control on water quality, and in particular, water quality improvement, approaches are required to assess aquifer microbial communities, their potential to augment treatment, response to changing geochemical conditions, and ultimately the sustainability of treatment. Leveraging the natural treatment capacity, where available, allows for the design of a sustainable treatment train and avoids overuse of energy-intensive engineered pretreatment without overtreating water prior to MAR—for example, a current environmental challenge is the widespread use and environmental impact of perfluoroalkyl and polyfluoroalkyl substances (PFAS). The fate of PFAS in MAR is uncertain and thus pretreatment or posttreatment technologies may be required to manage this risk (Page et al. 2019). Considering MAR as a step in a treatment train enables one to manage the complex topic of water quality when MAR alone cannot provide sufficient treatment, fate cannot be predicted, or where water quality degradation may occur.

The natural treatment processes endowed by storage in the aquifer are credited for helping the public to overcome the “yuck factor” associated with recycling treated wastewater for drinking water supply (Alley and Alley 2022). Faced with the unknowns and uncertainties of regulated and unregulated water quality threats, the assumption that storage time mitigates risk, especially of pathogens, biodegradable organic matter, and trace organic chemicals, is likely to hold (NRMMC, EPHC, NHMRC 2009), although more research is warranted to determine the time scale and environmental conditions (Hübner et al. 2022) for complete mineralization, including mostly unknown biotransformation byproducts (Ma et al. 2021). This should be of interest to many water banking authorities such as those in the western US states. The Kern Water Bank in the USA, initiated in the early 1970s, recharged 1.13 billion m^3^ through 44 km^2^ of recharge/spreading basins between 1995 and 2000 to alluvial fan deposits of the Kern River. Meillier et al. (2008) used dissolved chlorofluorocarbons (CFC-11 and CFC-12) to estimate groundwater ages of the target alluvial aquifer, finding that the youngest apparent ages (younger than 1985) were found in the shallow wells in the northern and central sections of the study area where MAR is usually performed. The recovered water here is suitable for irrigation but needs further treatment if used for drinking. With the new analytical capability regarding contaminants of emerging concerns and microbial genomes, water banking authorities, out of their fiduciary duty, can expand their monitoring programs to track the recharged water as it “ages” in the aquifer. For instance, it would be desirable to understand the storage time required under particular redox conditions to completely mineralize the myriad of trace organic contaminants.

## The way forward

To enhance climate resilience and other social, economic, and environmental benefits of groundwater through MAR, water quality threats from novel entities need to be addressed to maintain resource integrity. The aforementioned water quality challenges can be approached from a risk-based perspective grounded by precautionary principles, developed over time through practice to solve clogging issues, and overcome economic and policy barriers (Megdal et al. 2015). Strengthening institutional capacity for regulatory frameworks for water allocation, permit granting and water quality protection are especially relevant. It is important to balance the need to protect groundwater quality integrity (ecocentrism ethic) and the desire to use the natural treatment ability of the aquifer to improve water quality (anthropocentrism ethic). It is worth noting that when it comes to groundwater recharge laws in the United States, a communitarian ethic has been suggested to underpin regulatory processes (Owen 2021). Debate is encouraged on how to arrive at a sensible regulatory framework for MAR to manage water quality risks. Here, the perspective grounded in a communitarian ethic and the precautionary principle provides a starting point.

The Australian risk-based approach to MAR (NRMMC, EPHC, NHMRC 2009) is a model that sustainably protects groundwater quality, accounting for water quality changes, both improvements and deteriorations in the subsurface, and can be expanded geographically because many countries use the highly prescriptive approach of measuring compliance against uniform water quality parameters. In Europe, both the development and application of a legislative framework for MAR have varied among different countries, with current legislation ranging from strict and uniform water quality requirements to site-specific risk-based evaluation in the Netherlands (similar to Australia). A soon-to-be-effective European Union Directive 2020/741 has set minimum requirements for water quality, as well as monitoring and provisions on risk management applications for agricultural use of reclaimed water. A risk-based directive specific for MAR to further expand water reuse and recycling is a logical next step for the EU and any designated regulatory entities to consider.

The way forward clearly depends on regulations that value and enable the sustained use of natural treatment capacity provided by MAR, seamlessly integrated into a treatment train with pretreatment or posttreatment technologies as required. Advanced tools, including but not limited to real-time monitoring, data assimilation, and reactive-transport modeling, are required to predict the fate of chemicals and pathogens and to assess risks to human health and aquifer integrity. Currently, the natural attenuation or assimilatory capacity of aquifers has been relied upon for the degradation of many organic pollutants. As such, one could view this “attenuation zone” simultaneously as a subsurface natural treatment zone with a finite hydraulic retention time (Fig. 1). In addition to the determination of the hydraulic retention time, the understanding of the fate of pathogenic organisms, including attachment and inactivation and the variables that influence these, is necessary. Surrogates that can be used for laboratory and field verification, and genomic approaches for characterizing the health of subsurface microbial communities, also provide a broader perspective on the sustainability of microbial and trace organic removal processes. The IAH-MAR Commission strives to develop the body of scientific knowledge needed to have confidence in enhancing the sustainable and beneficial use of aquifers for humanity within the Earth’s safe operating space.
